# Research in Medicine: Its Need of Endowment and Outlook

**Published:** 1916-09-02

**Authors:** 


### RESEARCH IN MEDICINE.

### Its Need of Endowment and Outlook.

It is a commonplace truism that scientific research in general, like the teaching of science, is grossly underpaid in this country; and the generalisation holds good for medicine, amongst the other sciences, with a comparatively few exceptions. Fortunately for mankind, there is a type of mind, a species of human temperament, to which the desire of gain or the attainment of popular esteem is of very small moment compared with the pursuit of knowledge and the ascertainment of truth for its own sake. And it is largely to this circumstance that the profession of medicine and the community at large owe it that research takes place to the extent it does. It is true that private benefactors have here and there endowed research work to a certain degree: the Otto Beit Research Fellowships are the outstanding example of such munificence hitherto. But in spite of such occasional and exceptional instances of individual clearness of vision, it is true in the main that in our educational system scientific research is neglected and starved.

Here, then, is a very real reform waiting for those keen educationists who are busy just now with plans for improving our national system of education from top to bottom. The endowment of all scientific research is a very real need and a very difficult problem in some ways; and the endowment of medical research is perhaps as urgently needed as any other improvement in the whole realm of education. Hitherto medical research has been casual, unorganised, ill-paid. Many brilliant intellects have been attracted to it, but most of them have used it as a stepping-stone to material success as practising physicians or surgeons, and have dropped it as soon as the necessary "kudos" and advertisement have been gained. Only the men of true philosophic temperament—and they form but a small fraction of the men of ability and intellect in the profession—stick to research; and even then they can only do so, as a rule, if they chance to possess some private means. There are, no doubt, certain exceptions to this generalisation; that is, there are certain researchers who do keep on at research and do make it a business proposition. They are men who, like Lord Kelvin, possess the exceedingly rare power of scientific discovery and combine with it the gift for its utilitarian application. Such men achieve both distinction within the profession and reputation among the public: they "make a good thing" of research. But the tiny handful of them only shows how unusual this combination of gifts is: their names are household words, and there is no need to specify them here.

Research, then, as a rule is but ill-rewarded commercially in the medical profession. To those just entering on medical study the point is perhaps a trifle academic; for neither they nor their parents nor their schoolmasters can as yet have the slightest idea whether they are or are not likely to show aptitude for research; indeed, the chances are strongly against any given individual doing so. Boys do not begin medical study with the fixed idea of making research their career; so that the ill-endowment of research deters neither students nor their guardians from the embarkation of the former upon a medical curriculum. The endowment of research is advocated because the great majority of men with aptitude for such work and with ambitions to pursue it are prevented from so doing by considerations of bread and cheese, or attracted to other branches of medicine by the superior rewards there attainable.

In sustaining this view one must be careful to admit that there are exceptions to it. In a recently published book on the diagnosis of diseases of the heart* Sir James Mackenzie puts forward views which are quite opposed to it, but then Sir James is one of the exceptions—and a very brilliant one indeed—to the usual run of research workers. He not only carried on research of quite outstanding merit during many years of busy private practice in a provincial town; he has shown himself exceptional in another way too, by making fortune as well as fame out of his work. Sir James Mackenzie, in the book just mentioned, pleads for the carrying out of research by the general practitioner; nay, even by all general practitioners. If all practitioners possessed the patience, the energy, the philosophic breadth of mind, and the sheer intellect which he does, the recommendation might be feasible (though it would probably then be unnecessary). It is precisely because his combination of gifts is very rare that Sir James has so distinguished himself from among the general run of his contemporaries.

The selection of men for endowed posts as research workers is, of course, exceedingly difficult. Mere academic distinction alone is useless as a test, though certainly the majority of successful research workers are academically distinguished. Good degrees and gold medals no more make a man a philosopher than they make him a good operator; they are merely evidences of intelligence and industry, excellent and almost indispensable as foundations, but not in themselves sufficient. Probably selection by teachers is as good a ground plan as any, provided those teachers are entirely devoting themselves to teaching, or to teaching with research. No method of selection will pick the right men every time, for the simple reason that no test can estimate those inherent qualities of temperament and of intellect which form the equipment of the ideal research worker. Considerations of this sort can, however, wait; what cannot, and must not, wait is the recognition by educationists and by statesmen of the value of good research, and the pressing need of obtaining it by providing that those who take it up shall be the best obtainable for it, and shall be adequately rewarded.

---

* See review on p. 513.

